# Knowledge, Attitude and Practice towards the COVID-19 Pandemic: A Cross-Sectional Survey Study among the General Public in the Kingdom of Saudi Arabia

**DOI:** 10.3390/vaccines10111945

**Published:** 2022-11-17

**Authors:** Namdeo Prabhu, Meshal Aber Alonazi, Hmoud Ali Algarni, Rakhi Issrani, Sarah Hatab Alanazi, Mohammed Katib Alruwaili, Gharam Radhi Alanazi, Azhar Iqbal, Osama Khattak

**Affiliations:** 1 Department of Oral & Maxillofacial Surgery and Diagnostic Sciences, College of Dentistry, Jouf University, Sakaka 72388, Saudi Arabia; 2 Department of Conservative Dentistry, College of Dentistry, Jouf University, Sakaka 72388, Saudi Arabia; 3 Department of Preventive Dentistry, College of Dentistry, Jouf University, Sakaka 72388, Saudi Arabia; 4 Department of Periodontology and Endodontology, Faculty of Dental Medicine, Hokkaido University, N13W7, Kita-ku, Sapporo 060-8586, Hokkaido, Japan; 5 Department of Operative Dentistry & Endodontics, Frontier Medical and Dental College, Abbottabad 22010, Pakistan

**Keywords:** COVID-19, knowledge, pandemic, prevention, questionnaire

## Abstract

**Background:** The novel coronavirus disease 2019 (COVID-19) is an infectious disease that has been spreading worldwide in an unprecedented manner. The knowledge, attitude and practices of the general population play a vital role in prevention of COVID-19. **Objectives:** The present study aimed to assess the COVID-19-related knowledge, attitudes and practices of the general public of Sakaka, Saudi Arabia, to identify which populations show low levels of knowledge, attitudes and practices towards COVID-19, making them highly likely to remain vulnerable during the pandemic. **Methodology:** For this study, a nineteen-item closed-ended questionnaire was hand delivered to the general public, including patients and other hospital attendees attending the Outpatient Department of the College of Dentistry, Jouf University, Saudi Arabia. The research questions focused on the demographic information, knowledge, attitude and practices related to COVID-19. Data analysis is presented through tables and descriptive methods. **Results:** A total of 722 participants took part in the survey questionnaire. The majority of the respondents belonged to the age group of 28–37 years (n = 320; 44.3%), with female participants (n = 419; 58.0%) outnumbering the male participants (n = 303; 42.0%). Most of the respondents had good knowledge, attitudes and practices towards COVID-19. Patients aged 28–37 years (*p* = 0.000) with a master’s degree (*p* = 0.011) and government employees (*p* = 0.000) had significantly better knowledge than their counterparts. Significantly optimistic attitudes were noticed in participants aged 28–37 years (*p* = 0.000) with a master’s degree (*p* = 0.000), the married (*p* = 0.047) and government employees (*p* = 0.000). Government employees (*p* = 0.014) had significantly better practices. **Conclusions:** Overall, the participants of this study had good COVID-19-related knowledge, attitudes and practices. These findings would be useful in motivating the general population to follow the precautionary measures that will aid in prevention of COVID-19. Furthermore, the findings may help policymakers identify the target populations, especially the less educated and older adults, for COVID-19 prevention and health education.

## 1. Introduction

The novel coronavirus disease 2019 (COVID-19) was first discovered in China, in the city of Wuhan, and is alleged to have originated in bats and transmitted to humans by virtue of unknown hosts in the Wuhan seafood market in December 2019 [1]. The swift movement of people from global epicenters and amid the cities aided in rapid transmission and spread of the virus [2]. It was affirmed as a pandemic by the World Health Organization (WHO) on 11 March 2020 when more than 170 countries reported the presence of infections [3]. Coronavirus is a single-stranded RNA virus that belongs to the Orthocoronavirinae subfamily, which is from the family of Coronaviridae [4]. Most frequently reported symptoms are fever, cough, dyspnea and myalgia or fatigue. The chief modes of transmission include direct spread by coughing, sneezing and inhalation of droplets and contact transmission through contact with nasal, oral and ocular mucosa [5].

In an attempt to sever the spread of the disease, international travel deferment, contact tracing, containment and mitigation strategies were initiated by governments. The WHO also issued precise recommendations for the preclusion and control of COVID-19, such as the use of alcohol-based hand sanitizers, face masks, social distancing, self-isolation and medical attention for an infected person [6]. Additionally, lockdowns, referring to complete restriction on the movement, travel and non-essential activities, were implemented by different countries in an attempt to contain the spread [7]. The best way to prevent and slow down transmission is to be well informed about the COVID-19 virus, the disease it causes and how it spreads. Amidst pandemics, educating, engaging and mobilizing the public to become active participants may help achieve public health emergency preparedness, reducing the overall population’s vulnerability [8]. Previous studies on infectious disease epidemics have shown that knowledge, awareness, risk perception and efficacy belief has aided in motivating the general public to adopt preventive behaviors [8,9,10]. Similarly, for the COVID-19 pandemic, knowledge, perceived controllability, optimistic attitude and risk perception might aid in precautionary measures being taken by the public [11,12,13,14].

Knowledge, attitude and practice surveys are usually conducted to assess the knowledge gaps and behavioral patterns among socio-demographic subgroups, especially the vulnerable population, to implement or modify the public health interventions [15]. The issue of health inequalities noticed during disease outbreaks has been extensively investigated across pandemics [16,17]. For example, it was found that people with less education, with low income and living in more deprived neighborhoods were affected more during the novel influenza A (H1N1) outbreak [18,19,20]. During the 2015 Middle East Respiratory Syndrome (MERS) outbreak, social determinants were found to be directly (gender, education) or indirectly (age, education, income) related to practicing preventive behaviors [21]. Similarly, evidence of an unequal burden of COVID-19 is also emerging fast. It was found that the behavioral factors related to COVID-19 are unevenly distributed among people [22]. For example, prior studies have concluded that men, less educated individuals and the elderly had poor knowledge and behaviors towards COVID-19, and risk perception varied by the level of social support [8,23]. Given these alarming inequalities in behavioral factors, there remains an urgent need to identify vulnerable populations during the COVID-19 pandemic to ensure health education and communication interventions tailored to their needs [7].

There is limited evidence concerning behavioral factors and related vulnerability during the COVID-19 pandemic in Saudi Arabia. Therefore, the main purpose of this study was to assess the COVID-19-related knowledge, attitudes and practices of the general public of Sakaka, Saudi Arabia, to identify which populations show low levels of knowledge, attitudes and practices towards COVID-19, making them highly likely to remain vulnerable during the pandemic.

## 2. Materials and Methods

This cross-sectional survey study was conducted by using a validated self-administered questionnaire from 1 February 2022 to 31 March 2022 among the general public, including patients and other hospital attendees (e.g., the patient’s family members, relatives and friends) attending the Outpatient Department of the College of Dentistry, Jouf University, Sakaka, Saudi Arabia.

### 2.1. Sample Population, Size and Characteristics

Mean ± SD scores of the knowledge, attitude and practice regarding COVID-19 was calculated from the pilot study. Scoring was done separately for knowledge, attitude and practice and the highest mean ± SD score was considered. The representative target sample size needed, to achieve the study objectives and sufficient statistical power, was calculated with a sample size calculator [24]. The minimum sample required to detect a statistically significant difference of 10% in the mean scores of knowledge, attitude and practices, with a 95% confidence level and 80% power, was found to be 656. Anticipating 10% incomplete responses, a minimum of 700 were considered.

The participants were selected using the systematic random sampling technique after consideration of the inclusion and exclusion criteria. The inclusion criteria considered were (1) participants ≥18 years old; (2) understand English or Arabic language; (3) native residents of Sakaka; and (4) aware of the term “COVID-19”. Those who did not meet any of those criteria were excluded from the study. Ethical approval no. 03-02-42 was received from the Institutional Review Board before conducting the study.

### 2.2. Study Tool

The questionnaire was adapted from previous studies [25,26] that were used for other viral infections in different parts of the world. The questionnaire was modified, focusing on the COVID-19 pandemic, and few new statements were added for better understanding of the knowledge, attitude and practices of the local population towards COVID-19 (see Supplementary Information). The questions were clear, close-ended, easy to follow and not too long. The nineteen-item questionnaire was broadly categorized into four sections:

Section 1 comprised the participants’ background information, such as age, gender, qualification, marital status, etc. (Q1–Q5);

Section 2 of the questionnaire consisted of four major statements to evaluate knowledge regarding the nature of the COVID-19 disease and its mode of transmission. Knowledge regarding the cause of the disease, mode of transmission, and the risk factors associated with COVID was assessed. Respondents were requested to choose among three options provided: “Yes”, “No” or “Not Sure” (Q6–Q9);

Section 3 included eight major statements addressing public attitudes toward COVID-19 disease prevention, which included statements such as the use of hand wash and a well-fitting face mask, supporting COVID-19 patients and neighbors recovering from COVID-19, etc. Respondents were requested to choose among three options provided: “Yes”, “No” or “Not Sure” (Q10–Q17);

Section 4 comprised the two major statements regarding precautionary behavior practices that mainly focused on the preventive measures (e.g., maintaining adequate physical distancing, wearing facial masks, avoiding unnecessary travelling, etc.) and the reasons for wearing a face mask. Respondents were requested to choose among three options provided: “Always”, “Occasionally” or “Never” (Q18–Q19).

### 2.3. Procedure

The questionnaire was designed in two language versions: English and Arabic (the national language of Saudi Arabia). The English version of the questionnaire was developed initially and was then translated into Arabic. Face and content validation of the questionnaire was done by two senior faculty members who are expertise in survey research. Modifications were made based on feedback provided. A pilot study was conducted with 30 randomly selected subjects from the study site to assess the ability of the respondents to understand and answer the questionnaire provided. Reliability analysis demonstrated Cronbach’s alpha coefficients for this questionnaire ranging from 0.942 to 0.954 for different sections, which are considered relatively high and internally consistent. Systematic random sampling (first participant was selected through lottery method) was used for participant selection. The participation was made voluntary and the purpose of the survey was briefed to the respondents before obtaining a verbal informed consent. It was assured in the beginning of the questionnaire itself that the results of the survey would be only presented or published as aggregate data, maintaining the confidentiality of the participants’ personal information.

### 2.4. Measurements

Correct answers were assigned a score of one whereas incorrect or uncertain (do not know) responses were given a score of zero. The total score for knowledge ranged from 0 to 16, with higher scores indicating better COVID-19-related knowledge. The total score for attitude ranged from 0 to 9, with higher scores indicating better attitude. For practices, scores were calculated based on the respondents’ answers to each statement: 1 = strongly disagree; 2 = disagree; 3 = undecided; 4 = agree; and 5 = strongly agree. Scores were calculated by averaging the respondents’ answers to the twelve statements. Total scores ranged from 1 to 5, with higher scores indicating good and safe practices.

### 2.5. Statistical Analysis

Initially, descriptive statistics were calculated, followed by testing the statistical significance between the various variables. Univariate analysis was used to tabulate the frequency of the social and demographic statistics. An independent sample *t*-test was used to assess the differences in the mean knowledge, attitude and practice scores between genders. One-way analysis of variance (ANOVA) was used to assess the differences in the mean knowledge, attitude and practice scores for all other variables. SPSS software version 20.0 (IBM Corp., Armonk, NY, USA) was used for statistical analysis, with the significance level set as *p* ≤ 0.05.

## 3. Results

Eight hundred individuals responded to the survey, and after excluding individuals with missing data, 722 individuals were available for final analysis. The majority of the participants were between 28 and 37 years of age (n = 320; 44.3%). Females were the predominant participants (n = 419; 58.0%). The majority of the participants were bachelor’s degree holders (n = 468; 64.8%), married (n = 390; 54.0%), and were non-government employees (n = 185; 25.6%). A summary of the demographic characteristics of the participants is shown in Table 1.

### 3.1. Responses to Knowledge, Positive Attitude and Acceptable Practice Statements

The mean (±SD) COVID-19 knowledge, attitude and practice scores were 11.9 (±2.21), 6.7 (±1.18) and 4.6 (±0.34), respectively, as shown in Table 2. The knowledge, attitude and practice scores indicated that the participants had good knowledge, a positive attitude and acceptable practices regarding COVID-19.

Most of the participants had good knowledge about the disease. The majority of the candidates were aware that the infection is spread by a virus (n = 602; 83.4%). Modes of transmission for the spread of the virus were familiar among participants, including air/droplet spread, via an asymptomatic carrier, shaking hands with an infected person and from one person to another in the family. Regarding the people vulnerable to COVID-19, it was seen that majority of the participants responded to ‘people in crowded places’ (n = 699; 96.8%) followed by ‘senior citizens above 65 years of age’ (n = 628; 87.0%). Participants were aware about the availability of a vaccine/medicine in the market (n = 535; 74.1%) (Table 3).

The majority of the participants were positive about welcoming a neighbor recovering from COVID-19 (n = 543; 75.2%). The attitude pertaining to keeping information secret if a known person gets COVID-19 was mixed, as 339 (47.0%) respondents agreed to this, whereas 350 (48.5%) were not in favor. The majority (n = 630, 87.3%) of the participants were in favor of a contact case being self-isolated or quarantined for 14 days. Most of the participants were positive about supporting COVID-19 patients in an attempt to improve community health (n = 567; 78.5%). Regarding being concerned for getting COVID-19 infection, 443 (61.4%) participants marked ‘yes’. The majority (n = 655, 90.7%) of the participants highlighted that someone having cough or cold must cover his mouth and nose with a flexed elbow when coughing or sneezing. Additionally, 615 (85.2%) respondents were in favor of using a hand wash to curb the spread and 651 (90.2) respondents agreed that the use of well-fitting face mask is effective in preventing this illness (Table 4).

The majority of the participants agreed to safety measures to be advocated to prevent the spread of infection. Most of the respondents (n = 564; 78.1%) agreed to practice physical distancing at crowded places; 548 (75.9%) respondents believed in the concept of wearing a face mask; 478 (66.2%) respondents agreed to maintain at least 1 m distance between people; 486 (67.3) respondents believed in the concept of washing their hands with hand sanitizers; 474 (65.7%) respondents believed in avoiding touching their face; 480 (66.5%) respondents agreed to refraining from smoking and other activities that weaken the lungs; 444 (61.5%) respondents favored coughing into a flexed elbow; 373 (51.7) respondents agreed to avoid unnecessary travel and staying away from large groups of people; and 353 (48.9%) respondents favored incorporating a healthy diet (Table 5).

### 3.2. KAP Scores by Demographic Characteristics

Post-hoc pairwise comparison has identified that participants aged 28–37 years (*p* = 0.000) who had a master’s degree (*p* = 0.011) and were working in the government sector (*p* = 0.000) had significantly better knowledge as compared to others. Regarding attitude, for participants aged 28–37 years (*p* = 0.000), the married (*p* = 0.047), those with a master’s degree (*p* = 0.000) and government employees (*p* = 0.020) had a significantly better attitude as compared to others. Participants who were working in the government sector (*p* = 0.014) had significantly better practice as compared to others (Table 6).

## 4. Discussion

Owing to the novelty and ambiguity of COVID-19, a plethora of diverse information and implications were developed as precautionary measures to prevent this disease [27]. One of the precautionary measures implemented by most of the countries was a complete lockdown [28]. However, the decision to lockdown negatively affected the economy of the countries. The other problems faced by people globally included sleep disturbances, anxiety, depression, unemployment, decreased income, scarcity in food supply, etc. [29]. Despite the unparalleled national measures in fighting the outbreak, the success or failure depends on public behavior. Deeper insight into public discernment can be achieved by evaluating public awareness and knowledge about the disease, which may, in turn, persuade the general population in adopting healthy practices [30]. Therefore, the objective of this study was to assess the level of knowledge, attitudes and practices of Saudi residents towards COVID-19 and identify which populations have low levels of awareness towards COVID-19, making them highly likely to remain vulnerable during the pandemic.

Our findings reflected that the majority of the participants had thorough knowledge pertaining to the causative agent, modes of transmission, vulnerability to disease and availability of vaccination.

Similar findings were noted in studies done in different parts of the world, where participants have shown satisfactory levels of knowledge regarding COVID-19 [31,32]. On the contrary, the respondents of a study done in Iraq [33] and Bangladesh [34] had poor knowledge regarding COVID-19. The higher rate of good knowledge towards COVID-19 as noted in this study could be attributed to health authorities of Saudi Arabia that has been providing continuous awareness to the public through recurring messages on mobile phones and innovative ideas to catch the attention of denizens. Studies from China [14], Saudi Arabia [31] and Iraq [33] have shown better knowledge among participants with a higher academic degree and over the age of 30 years. In the current study, participants aged 28–37 years and with a master’s degree had statistically better knowledge as compared to their counterparts. It could be that people with a high level of education are eager to collect information more than others. Additionally, less educated people cannot communicate with others easily, and also, they might face difficulty in using social media for tracking news. Therefore, this study suggests that a targeted educational program should be directed to the less educated and young people to improve knowledge on the COVID pandemic. It is worth noting that, in this study, none of the participants were above the age of 68 years. This could be attributed to the short duration of the study and also due to the fact that usually the older adults are accompanied by relatives or caretakers who are mostly young adults that might have responded to the survey questionnaire. The patients who are old generally come for short dental appointments owing to their ageing problems. Surprisingly, in this study, the male and female participants had an almost similar response regarding the knowledge statements, in contrast to previous studies, where women had better knowledge about COVID-19 compared to men [12,31,32]. Additionally, there was no significant difference in marital status of a participant regarding knowledge. In contrast, a study done among the Saudi population regarding self-isolation during the COVID-19 pandemic had revealed that unmarried and widowed participants were more willing to self-isolate as compared to married individuals [35].

The study conducted by Ali Jadoo SA et al. showed a high attitude score towards COVID-19 among the Syrian people resident in Turkey [32]. On the other hand, the study done by Issrani R et al. in Saudi Arabia found that most of the respondents had an average attitude to willingly self-isolate during the COVID-19 pandemic [35]. The current study found that a large majority of participants had positive attitudes toward COVID-19 illness. The majority of the participants were in favor of a contact case being self-isolated or quarantined for 14 days; they also highlighted the importance of covering the mouth and nose with a flexed elbow when coughing or sneezing, use a hand wash to curb the spread, and agreed to the use of a well-fitting face mask in preventing the illness. These findings prove that Saudi residents are very cautious and follow the precautionary measures against COVID-19 infection. Regarding the statement ‘keeping the information secret if any of your known gets COVID-19’, the participants of this study gave equal responses for the options ‘yes’ and ‘no’. This finding is inconsistent with another study done in Saudi Arabia in which the respondents strongly agreed in letting the neighbors or colleagues know about an individual being positive for COVID infection [35]. The same study attributed this behavior either due to individuals’ fear that they might spread the disease to their closed ones or could be due to the possible social pressures because anyone who does not follow such normative measures is treated as a public enemy [35]. Additionally, our results showed that participants aged 28–37 years, married, with a master’s degree and government employees had a significantly better attitude towards COVID-19 as compared to their counterparts.

The study done among Saudi public showed that the respondents followed good practices for majority of the statements other that when and whom wearing masks to prevent infection [31]. The participants of the present study adopted good and safe practices related to COVID-19 and almost all of the participants knew the reasons for wearing a mask. This finding could be attributed to the great deal of efforts of Saudi Arabia’s health authorities in providing education and outreach materials and increasing the public understanding of the disease.

The current COVID-19 pandemic and other infectious disease outbreaks provide an opportunity to emphasize the importance of adherence to published precautionary strategies to combat the disease. It is worth noting that the health authorities of Saudi Arabia have done a great deal of efforts at all levels, including public awareness campaigns. The Saudi Ministry of Health (MOH) has conducted an intensive awareness-raising campaign, communicated via its website, television and various social media. The guide with precautionary measures against COVID-19 has been provided by the MOH in more than 10 languages. It also interacted with the public and the media, especially via social media platforms. Furthermore, the government of Saudi Arabia has launched smartphone applications (namely, Tetamman, Tabaud and Tawakkalna) to prevent the spread of COVID-19, provide information about the number of coronavirus infections in the kingdom and early detection of coronavirus infections once users show COVID-19 symptoms [36]. These early interventions on engaging the general population in preventive measures, as well as efforts to combat rumors and misinformation, have been greatly expanded [37,38]. The unique and recent experience of Saudi Arabia in dealing successfully with two viral outbreaks has unquestionably helped the government in taking a prompt response and precautionary measures against COVID-19 to control its spread [39,40]. Although the authorities in Saudi Arabia have left no stone unturned to achieve these goals, the findings of the current study identified a vulnerable population that needs more encouragement to follow precautionary measures during a pandemic situation.

### 4.1. Implications of the Study

This study was undertaken to enhance the awareness of the general public of the northern part of Saudi Arabia towards COVID-19 and to identify the vulnerable population that needs more attention and encouragement for the prevention of COVID-19-related illnesses. Although the Saudi government has managed to prevent the spread of COVID-19 infection by continuous creation of awareness amongst the community and by imposing an early phased lockdown, the findings of the present study has identified vulnerable populations, such as the less educated and older adults, that need more encouragement to curb the spread of the novel coronavirus.

### 4.2. Limitations

The present study has some limitations. Similar to all self-administered public surveys, the accuracy of the results was heavily dependent on the honesty and understanding of the respondents. Furthermore, all the data were from one city in the northern part of Saudi Arabia; thus, the findings may not be applicable for populations outside this geographic area and so generalizations of the findings are limited.

## 5. Conclusions

This study provides a timely and meaningful understanding of the level of the public’s knowledge, attitude and practices regarding the COVID-19 pandemic. Our findings suggest that Saudi residents, especially the young and well-educated, have good knowledge, optimistic attitudes and safe practices towards COVID-19. This study recommends that more attention should be given to the less educated and older adults to follow the standard precautionary measures. Governments should provide advanced and motivational education methods to educate the vulnerable population for possible future pandemics. Furthermore, the findings of this study might offer a significant reference point for follow-up longitudinal studies to assess an individual’s behavior towards COVID-19.

## Figures and Tables

**Table 1:** Summary of the demographic characteristics.

Variable	Number (N = 722)	Percentage (%)
**Age**		
18–27	221	30.6
28–37	320	44.3
38–47	157	21.7
48–57	16	2.2
58–67	08	1.1
≥68	00	00
**Gender**		
Male	303	42.0
Female	419	58.0
**Education**		
Primary school and below (up to grade 6 and below)	15	2.1
Secondary school (Grade 7 to grade 12)	85	11.8
Bachelor	468	64.8
Diploma	95	13.2
Master	26	3.6
PhD	33	4.6
**Marital status**		
Not married	309	42.8
Married	390	54.0
Divorced	21	2.9
Widowed	2	0.3
**Occupation**		
Self-employed	103	14.3
Non-government employee	185	25.6
Government employee	03	0.4
Retired	149	20.6
Student	134	18.6
Unemployed	148	20.5

**Table 2:** Mean knowledge, attitude and practice scores.

Variable	Mean	SD	Minimum	Maximum
Knowledge score	11.9	2.21	3	16
Attitude score	6.7	1.18	3	9
Practice score	4.6	0.34	1	5

**Table 3:** Responses to the questionnaire on COVID-19 knowledge.

Statement	N (%)		
	Yes	No	Not Sure
**COVID-19 illnesses are caused by:**			
Viruses	602 (83.4)	108 (15.0)	12 (1.7)
Bacteria/Mosquitoes	249 (34.5)	420 (58.2)	53 (7.3)
**COVID-19 illness spreads by:**			
Air/droplets	484 (67.0)	110 (15.2)	128 (17.7)
Blood	321 (44.5)	251 (34.8)	150 (20.8)
Water	393 (54.4)	207 (28.7)	122 (16.9)
COVID-19 carriers can transmit the infection	649 (89.9)	64 (8.9)	09 (1.2)
Through a person with NO symptoms	607 (84.1)	64 (8.9)	51 (7.1)
Sharing towels with an infected person	606 (83.9)	92 (12.7)	24 (3.3)
Shaking the hands of an infected person with a cough and/or cold	635 (88.0)	42 (5.8)	45 (6.2)
From one person to another in the family	659 (91.3)	43 (6.0)	20 (2.8)
**The following persons are at an increased risk of COVID-19 illness:**			
Senior citizens aged 65 years and older	628 (87.0)	44 (6.1)	50 (6.9)
Smokers	505 (69.9)	105 (14.5)	112 (15.5)
Asthmatics	575 (79.6)	69 (9.6)	78 (10.8)
Diabetes	501 (69.4)	113 (15.7)	108 (15.0)
Those in crowded places/among a lot of people	699 (96.8)	14 (1.9)	9 (1.2)
**Is there any vaccine/medicine available in market to cure COVID-19 illness?**	535 (74.1)	118 (16.3)	69 (9.6)

**Table 4:** Responses to attitudinal statements regarding COVID-19.

Statement	N (%)		
	Yes	No	Not Sure
Welcome a neighbor recovering from COVID-19	543 (75.2)	157 (21.7)	22 (3.0)
Keep information secret if any of your known gets COVID-19	339 (47.0)	350 (48.5)	33 (4.6)
A contact case be quarantine/self-isolate for 14 days	630 (87.3)	71 (9.8)	21 (2.9)
Supporting COVID-19 patients improves community health	567 (78.5)	65 (9.0)	90 (12.5)
I am concerned of being infected with COVID-19	443 (61.4)	256 (35.5)	23 (3.2)
I feel that someone who has cold/cough should- cover his mouth and nose with his bare hand when coughing or sneezing cover his mouth and nose with a flexed elbow when coughing or sneezing	418 (57.9) 655 (90.7)	273 (37.8) 54 (7.5)	31 (4.3) 13 (1.8)
Using a hand wash can prevent this illness	615 (85.2)	66 (9.1)	41 (5.7)
Wearing a well-fitting face mask is effective in preventing this illness	651 (90.2)	58 (8.0)	12 (1.8)

**Table 5:** Practices related to COVID-19.

Statement	N (%)				
	Strongly Disagree	Disagree	Neutral	Agree	Strongly Agree
**The following practices can help protect you from COVID-19 illness:**					
Ensuring a healthy diet	13 (1.8)	16 (2.2)	70 (9.7)	270 (37.4)	353 (48.9)
Maintain at least 1 m distance between you and people coughing or sneezing	7 (1.0)	6 (0.8)	17 (2.4)	214 (29.6)	478 (66.2)
Washing your hands with hand sanitizers	24 (3.3)	13 (1.8)	16 (2.2)	183 (25.3)	486 (67.3)
Wearing a face mask	00 (0.0)	00 (0.0)	10 (1.4)	164 (22.7)	548 (75.9)
Coughing into a flexed elbow	6 (0.8)	8 (1.1)	23 (3.2)	241 (61.5)	444 (61.5)
Avoid touching your face	17 (2.4)	14 (1.9)	24 (3.3)	193 (26.7)	474 (65.7)
Refrain from smoking and other activities that weaken the lungs	28 (3.9)	23 (3.2)	36 (5.0)	155 (21.5)	480 (66.5)
Practice physical distancing at crowded places	01 (0.1)	12 (1.7)	31 (4.3)	114 (15.8)	564 (78.1)
Unnecessary travel and staying away from large groups of people	08 (1.1)	15 (2.1)	174 (24.1)	152 (21.1)	373 (51.7)
**The following are reasons for wearing a mask:**					
Being in crowded places	00 (0.0)	00 (0.0)	11 (1.5)	97 (13.4)	614 (85.0)
Being near people who are coughing	00 (0.0)	00 (0.0)	25 (3.5)	108 (15.0)	589 (81.6)
When I am sick	00 (0.0)	00 (0.0)	33 (4.6)	111 (15.4)	578 (80.1)

**Table 6:** Comparison of the mean KAP score and demographic characteristics.

Variable	N	%	Knowledge Score			Attitude Score			Practice Score		
			Mean	SD	*p*	Mean	SD	*p*	Mean	SD	*p*
**Age**											
18–27	221	30.6	11.4	2.15	0.000	6.3	1.09	0.000	4.6	0.34	0.437
28–37	320	44.3	12.2	2.23		7.0	1.20		4.6	0.34	
38–47	157	21.7	12.2	2.18		6.8	1.10		4.5	0.33	
48–57	16	2.2	11.9	1.91		6.9	1.31		4.6	0.25	
58–67	08	1.1	14.0	0.00		7.0	0.00		4.7	0.15	
≥68	00	00	--	--		--	--		--	--	
**Gender**											
Male	303	42.0	11.9	2.21	0.371	6.7	1.15	0.523	4.6	0.37	0.225
Female	419	58.0	12.0	2.21		6.8	1.20		4.6	0.31	
**Education**											
Primary school and below (up to grade 6 and below)	15	2.1	12.3	1.98	0.011	7.1	0.35	0.000	4.7	0.19	0.085
Secondary school (Grade 7 to grade 12)	85	11.8	11.9	1.75		6.7	0.86		4.6	0.32	
Bachelor	468	64.8	12.0	2.25		6.7	1.27		4.6	0.34	
Diploma	95	13.2	11.7	2.33		6.5	0.92		4.6	0.31	
Master	26	3.6	13.3	2.18		7.7	1.42		4.7	0.22	
PhD	33	4.6	11.2	2.13		7.1	0.70		4.6	0.37	
**Marital status**											
Not married	309	42.8	12.1	2.16	0.332	6.6	1.14	0.047	4.6	0.33	0.133
Married	390	54.0	11.9	2.29		6.8	1.22		4.6	0.33	
Divorced	21	2.9	11.7	1.11		6.5	0.60		4.4	0.41	
Widowed	2	0.3	12.5	2.12		6.5	0.71		4.8	0.28	
**Occupation**											
Self-employed	103	14.3	11.3	2.06	0.000	6.4	1.12	0.020	4.5	0.36	0.014
Non-government employee	185	25.6	12.4	2.34		6.7	1.26		4.5	0.39	
Government employee	0.3	0.4	13.3	2.08		7.3	0.58		4.8	0.2	
Retired	149	20.6	12.3	2.33		6.7	1.32		4.6	0.29	
Student	134	18.6	11.6	1.95		6.8	0.89		4.7	0.26	
Unemployed	148	20.5	11.9	2.11		7.0	1.16		4.6	0.32	

## Data Availability

The data set used in the current study will be made available on request from Namdeo Prabhu; dr.namdeo.prabhu@jodent.org.

## Supplementary Materials

The following supporting information can be downloaded at: https://www.mdpi.com/article/10.3390/vaccines10111945/s1, The survey questionnaire is available at the Supplementary Materials.

## References

[1] Alanagreh L., Alzoughool F., Atoum M. (2020). The human coronavirus disease COVID-19: Its origin, characteristics, and insights into potential drugs and its mechanisms. Pathogens.

[2] Bashir T.F., Hassan S., Maqsood A., Khan Z.A., Issrani R., Ahmed N., Bashir E.F. (2020). The psychological impact analysis of novel COVID-19 pandemic in health sciences students: A global survey. Eur. J. Dent..

[3] Guo Y.R., Cao Q.D., Hong Z.S., Tan Y.Y., Chen S.D., Jin H.J., Tan K.-S., Wang D.-Y., Yan Y. (2020). The origin, transmission and clinical therapies on coronavirus disease 2019 (COVID-19) outbreak—An update on the status. Mil. Med. Res..

[4] Yang D., Leibowitz J.L. (2015). The structure and functions of coronavirus genomic 3′ and 5′ ends. Virus Res..

[5] Jayaweera M., Perera H., Gunawardana B., Manatunge J. (2020). Transmission of COVID-19 virus by droplets and aerosols: A critical review on the unresolved dichotomy. Environ. Res..

[6] Mustafa R.M., Alrabadi N.N., Alshali R.Z., Khader Y.S., Ahmad D.M. (2020). Knowledge, attitude, behavior, and stress related to COVID-19 among undergraduate health care students in Jordan. Eur. J. Dent..

[7] Lee M., Kang B.A., You M. (2021). Knowledge, attitudes, and practices (KAP) toward COVID-19: A cross-sectional study in South Korea. BMC Public Health.

[8] Lee M., You M. (2020). Psychological and behavioral Responses in South Korea during the early stages of coronavirus disease 2019 (COVID-19). Int. J. Environ. Res. Public Health.

[9] Aburto N.J., Pevzner E., Lopez-Ridaura R., Rojas R., Lopez-Gatell H., Lazcano E., Hernandez-Avila M., Harrington T.A. (2010). Knowledge and adoption of community mitigation efforts in Mexico during the 2009 H1N1 pandemic. Am. J. Prev. Med..

[10] Anderson R.M., Heesterbeek H., Klinkenberg D., Hollingsworth T.D. (2020). How will country-based mitigation measures influence the course of the COVID-19 epidemic?. Lancet.

[11] Rahman A., Sathi N.J. (2020). Knowledge, attitude, and preventive practices toward COVID-19 among Bangladeshi internet users. Elect. J. Gen Med..

[12] Azlan A.A., Hamzah M.R., Sern T.J., Ayub S.H., Mohamad E. (2020). Public knowledge, attitudes and practices towards COVID-19: A cross-sectional study in Malaysia. PLoS ONE.

[13] Honarvar B., Lankarani K.B., Kharmandar A., Shaygani F., Zahedroozgar M., Rahmanian Haghighi M.R., Ghahramani S., Honarvar H., Daryabadi M.M., Salavati Z. (2020). Knowledge, attitudes, risk perceptions, and practices of adults toward COVID-19: A population and field-based study from Iran. Int. J. Public Health.

[14] Zhong B.L., Luo W., Li H.M., Zhang Q.Q., Liu X.G., Li W.T., Li Y. (2020). Knowledge, attitudes, and practices towards COVID-19 among Chinese residents during the rapid rise period of the COVID-19 outbreak: A quick online cross-sectional survey. Int. J. Biol. Sci..

[15] Papagiannis D., Malli F., Raptis D.G., Papathanasiou I.V., Fradelos E.C., Daniil Z., Rachiotis G., Gourgoulianis K.I. (2020). Assessment of knowledge, attitudes, and practices towards new coronavirus (SARS-CoV-2) of health care professionals in Greece before the outbreak period. Int. J. Environ. Res. Public Health.

[16] Bambra C., Riordan R., Ford J., Matthews F. (2020). The COVID-19 pandemic and health inequalities. J. Epidemiol. Community Health.

[17] Marmot M., Allen J. (2020). COVID-19: Exposing and amplifying inequalities. J. Epidemiol. Community Health.

[18] Lowcock E.C., Rosella L.C., Foisy J., McGeer A., Crowcroft N. (2012). The social determinants of health and pandemic H1N1 2009 influenza severity. Am. J. Public Health.

[19] Rutter P.D., Mytton O.T., Mak M., Donaldson L.J. (2012). Socio-economic disparities in mortality due to pandemic influenza in England. Int. J. Public Health.

[20] Biggerstaff M., Jhung M.A., Reed C., Garg S., Balluz L., Fry A.M., Finelli L. (2014). Impact of medical and behavioural factors on influenza-like illness, healthcare-seeking, and antiviral treatment during the 2009 H1N1 pandemic: USA, 2009–2010. Epidemiol. Infect..

[21] Lee M., Ju Y., You M. (2020). The effects of social determinants on public health emergency preparedness mediated by health communication: The 2015 MERS outbreak in South Korea. Health Commun..

[22] Lee L.Y., Lam E.P., Chan C.K., Chan S.Y., Chiu M.K., Chong W.H., Chu K.-W., Hon M.-S., Kwan L.-K., Tsang K.-L. (2020). Practice and technique of using face mask amongst adults in the community: A cross-sectional descriptive study. BMC Public Health.

[23] Lau L.L., Hung N., Go D.J., Ferma J., Choi M., Dodd W., Wei X. (2020). Knowledge, attitudes and practices of COVID-19 among income-poor households in the Philippines: A cross-sectional study. J. Glob. Health.

[24] http://www.raosoft.com/samplesize.html.

[25] Lal D., Sidhu T.K., Coonar P.P.S., Kaur H. (2016). An interventional study to assess knowledge and attitude of medical students towards ebola virus disease. Int. J. Com. Health Med. Res..

[26] Abdela A., Woldu B., Haile K., Mathewos B., Deressa T. (2016). Assessment of knowledge, attitudes and practices toward prevention of hepatitis B virus infection among students of medicine and health sciences in Northwest Ethiopia. BMC Res. Notes.

[27] Lamba I. (2020). Why India needs to extend the nationwide lockdown. Am. J. Emerg. Med..

[28] Chan J.F., Yuan S., Kok K.H., To K.K., Chu H., Yang J., Xing F., Liu J., Yip C.C.-Y., Poon R.W.-S. (2020). A familial cluster of pneumonia associated with the 2019 novel coronavirus indicating person-to-person transmission: A study of a family cluster. Lancet.

[29] Sahin A.R., Erdogan A., Agaoglu P.M., Dineri Y., Cakirci A., Senel M., Okyay R.A., Tasdogan A.M. (2020). 2019 Novel coronavirus (COVID-19) outbreak: A review of the current literature. EJMO.

[30] Cao J., Tu W.J., Cheng W., Yu L., Liu Y.K., Hu X., Liu Q. (2020). Clinical features and short-term outcomes of 102 patients with coronavirus disease 2019 in Wuhan, China. Clin. Infect. Dis..

[31] Al-Hanawi M.K., Angawi K., Alshareef N., Qattan A.M.N., Helmy H.Z., Abudawood Y., Al Qurashi M., Kattan W., Kadasah N.A., Chirwa G.C. (2020). Knowledge, attitude and practice toward COVID-19 among the public in the Kingdom of Saudi Arabia: A cross-sectional study. Front. Public Health.

[32] Ali Jadoo S.A., Dastan I., Al-Samarrai M.A.M., Yaseen S.M., Abbasi A., Alkhdar H., Al Saad M., Danfour O.M. (2020). Knowledge, attitude, and practice towards COVID-19 among Syrian people resident in Turkey. J. Ideas Health.

[33] Ali Jadoo S.A., Alhusseiny A., Yaseen S., Al-Samarrai M., Al-Rawi R., Al-Delaimy A., Abed M.W., Hassooni H.R. (2020). Knowledge, attitude, and practice toward COVID-19 among Iraqi people: A web-based cross-sectional study. J. Ideas Health.

[34] Ferdous M.Z., Islam M.S., Sikder M.T., Mosaddek A.S.M., ZegarraValdivia J.A., Gozal D. (2020). Knowledge, attitude, and practice regarding COVID-19 outbreak in Bangladesh: An online-based crosssectional study. PLoS ONE.

[35] Issrani R., Alam M.K. (2021). Theory of planned behavior as a conceptual framework for the willingness to self-isolate during the COVID-19 pandemic: A regional cross-sectional study. Work.

[36] Hidayat-ur-Rehman I., Ahmad A., Ahmed M., Alam A. (2021). mobile applications to fight against COVID-19 pandemic: The case of Saudi Arabia. TEM J..

[37] WHO (2020). WHO, Saudi Arabia Join Forces to Fight COVID-19 Nationally, Regionally and Globally. http://www.emro.who.int/media/news/who-saudi-arabia-join-forces-to-fight-covid-19-nationallyregionally-and-globally.html.

[38] Prabhu N., Issrani R. (2020). Dilemma in the foreign lands during COVID-19: An outlook of expat health care professionals with a take on a social perspective. Work.

[39] Madani T.A. (2004). Preventive strategies to keep Saudi Arabia SARS-free. Am. J. Infect. Control..

[40] Al Shehri A.M. (2015). A lesson learned from Middle East respiratory syndrome (MERS) in Saudi Arabia. Med. Teach..

