# Supplementary material for: Knowledge, Attitude and Practice towards the COVID-19 Pandemic: A Cross-Sectional Survey Study among the General Public in the Kingdom of Saudi Arabia
Source: Vaccines (Basel). 2022 Nov 17;10(11):1945. doi: 10.3390/vaccines10111945 (PMC9695995; doi:10.3390/vaccines10111945)
Supplement: Supplementary file 1 [file vaccines-10-01945-s001.zip › vaccines-1940591-supplementary.pdf]

SURVEY ON COVID-19 DISEASE

DIRECTIONS: The information you supply on this questionnaire will be kept confidential. The data will be used for research purposes.

Mark the single most appropriate response in each case. If, however, any question requests information that you do not wish to provide, feel free to omit it.

SECTION I- BACKGROUND INFORMATION

1. What is your gender?

a) Male   b) Female

2. How old are you?

a) 18 - 27   b) 28 - 37   c) 38 - 47   d) 48 - 57   e) 58 – 67   f) Older than 68

3. What is your highest level of education?

a) Primary school and below (Upto grade 6 and below)
b) Secondary school (Grade 7 to grade 12)
c) Bachelor
d) Diploma
e) Master
f) PhD

4. Are you married?

a) No   b) Yes   c) Divorced   d) Widowed

5. What is your occupation?

a) Self-employed   b) Non-government employee   c) Government employee
d) Retired   e) Student   e) Unemployed

SECTION II- KNOWLEDGE REGARDING THE NATURE OF DISEASE & ITS MODES OF TRANSMISSION

Item Number	Statement	Yes	No	Not sure
6	**COVID-19 illnesses are caused by-**			
	i. Viruses			
	ii. Bacteria/Mosquitoes			
7	**COVID-19 illnesses spreads by-** i. Air/droplets			
	ii. Blood			
	iii. Water			
	iv. COVID-19 carriers can transmit the infection			
	v. Through a person with NO symptoms			
	vi. Sharing towels with an infected person			
	vii. Shaking the hands of an infected person with a cough and/or cold			

	viii. From one person to another in the family			
8	**The following persons are at an increased risk of COVID-19 illnesses-** i. Senior citizens aged 65 and older			
	ii. Smokers			
	iii. Asthmatics			
	iv. Diabetes			
	v. Those in crowded places/among a lot of people			
9	**Is there any vaccine/medicine available in market to cure COVID-19 illnesses?**			

SECTION III - ATTITUDE TOWARD COVID-19 DISEASE PREVENTION

Item Number	Statement	Yes	No	Not sure
10	**Welcome a neighbor recovering from COVID-19**			
11	**Keep information secret if any of your known gets COVID-19**			
12	**A contact case be quarantine/self-isolate for 14 days**			
13	**Supporting COVID-19 patients improves community health**			
14	**I have no concern of being infected with COVID-19**			
15	**I feel that someone who has cold/cough should-** **i. cover his mouth and nose with his bare hand when coughing or sneezing** **ii. cover his mouth and nose with a flexed elbow when coughing or sneezing**			
16	**Using a hand wash can prevent this illness**			
17	**Wearing a well-fitting face mask is effective in preventing this illness**			

SECTION IV – PRACTICE RELATED TO COVID-19 PREVENTION

Item Number	Items on Plagiarism	Always	Occasionally	Never
18	**The following practices can help protect you from COVID-19 illnesses-**			
	i. Ensuring a healthy diet			
	ii. Maintain at least 1 metre distance between you and people coughing or sneezing			
	iii. Washing your hands with hand sanitizers			
	iv. Wearing a face mask			
	v. Coughing into a flexed elbow			
	vi. Avoid touching your face			
	vii. Refrain from smoking and other activities that weaken the lungs			
	viii. Practice physical distancing by avoiding			

	ix. Unnecessary travel and staying away from large groups of people			
19	**The following are reasons for wearing a mask-**			
	i. Being in crowded places			
	ii. Being near people who are coughing			
	iii. When I am sick			

Thank you for taking the time to respond thoughtfully to these questions
